# Prevalence, treatment, and attributed mortality of elevated blood pressure among a nationwide population-based cohort of stroke survivors in China

**DOI:** 10.3389/fcvm.2022.890080

**Published:** 2022-09-30

**Authors:** Bin Wang, Xueke Bai, Yang Yang, Jianlan Cui, Lijuan Song, Jiamin Liu, Jiapeng Lu, Jun Cai

**Affiliations:** ^1^National Clinical Research Center for Cardiovascular Diseases, National Health Commission of People’s Republic of China (NHC) Key Laboratory of Clinical Research for Cardiovascular Medications, State Key Laboratory of Cardiovascular Disease, Fuwai Hospital, Chinese Academy of Medical Sciences and Peking Union Medical College, National Center for Cardiovascular Diseases, Beijing, China; ^2^Hypertension Center, State Key Laboratory of Cardiovascular Disease, Chinese Academy of Medical Sciences and Peking Union Medical College, Fuwai Hospital, Beijing, China

**Keywords:** stroke, hypertension, mortality, epidemiology, prevention

## Abstract

**Background:**

Elevated blood pressure (BP) is associated with substantial morbidity and mortality in stroke survivors. China has the highest prevalence of stroke survivors and accounts for one-third of stroke-related deaths worldwide. We aimed to describe the prevalence and treatment of elevated BP across age, sex, and region, and assess the mortality attributable to elevated BP among stroke survivors in China.

**Materials and methods:**

Based on 3,820,651 participants aged 35–75 years from all 31 provinces in mainland China recruited from September 2014 to September 2020, we assessed the prevalence and treatment of elevated BP (systolic BP ≥ 140 mmHg or diastolic BP ≥ 90 mmHg) among those with self-reported stroke and stratified by age group, sex, and geographic region. We estimated the age- and sex-specific population attributable fractions of death from elevated BP.

**Results:**

Among 91,406 stroke survivors, the mean (SD) age was 62 (8) years, and 49.0% were male. The median interquartile range (IQR) stroke duration was 4 (2, 7) years. The prevalence of elevated BP was 61.3% overall, and increased with age (from 47.5% aged 35–44 years to 64.6% aged 65–75 years). The increment of prevalence was larger in female patients than male patients. Elevated BP was more prevalent in northeast (66.8%) and less in south (54.3%) China. Treatment rate among patients with elevated BP was 38.1%, and rates were low across all age groups, sexes, and regions. Elevated BP accounted for 33 and 21% of cardiovascular and all-cause mortality among stroke survivors, respectively. The proportion exceeded 50% for cardiovascular mortality among patients aged 35–54 years.

**Conclusion:**

In this nationwide cohort of stroke survivors from China, elevated BP and its non-treatment were highly prevalent across all age groups, sexes, and regions. Elevated BP accounted for nearly one-third cardiovascular mortality in stroke survivors, and particularly higher in young and middle-aged patients. National strategies targeting elevated BP are warranted to address the high stroke burden in China.

## Introduction

Stroke leads to 10% of all deaths (1) and nearly 5% of all disability-adjusted life years globally (2). China had 13 million prevalent stroke cases in 2018 (3) and one-third of stroke-related mortality worldwide (4). Elevated blood pressure (BP) is the most important modifiable risk factor in both primary and secondary prevention of stroke; BP control effectively reduces the risk of recurrent stroke and mortality among stroke survivors (5, 6). Improving outcomes in stroke survivors by optimizing BP management has been a national healthcare priority in China (7).

Despite the importance of BP control in secondary stroke prevention, little is known about the burden of elevated BP as well as its attributed mortality among community-dwelling stroke survivors in China, a vast country with a large population and geographic diversity. Previous studies of stroke survivors were mostly conducted among patients from particular cities or hospital-based populations with small sample size. In one cross-sectional survey in 2008 including 2,283 outpatient clinics from secondary and tertiary hospitals, half of patients with ischemic stroke (IS) or transient ischemic attack (TIA) did not achieve BP control (8). Two small sample studies conducted in primary healthcare sites in 2008 (9) and 2013 (10) reported the prevalence of elevated BP in IS survivors with hypertension was 71.9% (532/740) and 84.6% (220/260). Given the regional and urban-rural epidemiologic distinctions of stroke in China, uncertainty remains about the current status of BP management among stroke survivors by age, sex, and geographic region. Furthermore, the contribution of elevated BP to the cardiovascular (CV) and total mortality among stroke survivors is not well understood. Thus, a nationwide study on the prevalence, treatment of elevated BP, as well as its impact on mortality among stroke survivors is required to provide insights into the gaps for secondary prevention and herald directions for future interventions.

Accordingly, using data from the China Patient-centred Evaluative Assessment of Cardiac Events Million Persons Project (PEACE MPP), we aim to describe the prevalence and medication treatment of elevated BP among stroke survivors by age, sex, and geographic region, and assess the age- and sex-specific impact of elevated BP on CV and all-cause mortality among stroke survivors.

## Materials and methods

### Study participants and design

The China PEACE MPP is a national, population-based screening project funded by the Chinese government to identify individuals at high risk of CV disease for intervention and long-term management. Details of the project have been previously published (11). From September 2014 to September 2020, we applied a convenience sampling strategy to select 299 sites (180 rural counties, 119 urban districts) from all 31 provinces in mainland China. For each province, approximately eight counties or districts were selected using a typical case sampling design to provide diversity in geographical distribution, economic development, and population structure. The inclusion criteria were: (1) age 35–75 years, and (2) living in the region for at least 6 of the preceding 12 months. Potential participants were invited to the study by local community workers *via* extensive publicity campaigns on television and in newspapers. Individuals were asked to go into clinics to undergo measurements, including weight, height, BP, serum lipid level, and blood glucose level. A questionnaire was conducted to collect information on sociodemographic information, medical history, and current medications. The central ethics committee at the China National Center for Cardiovascular Diseases approved this project. All enrolled participants provided written informed consent.

### Data collection and definitions

For each participant, BP, blood lipids, height, and weight were measured according to standardized protocol. BP was measured using an electronic BP monitor (Omron HEM-7430; Omron Corporation, Kyoto, Japan) placed on the right upper arm with patient in seated position. Two readings were taken, 1 min apart. If the difference between the two systolic BP (SBP) measurements exceeded 10 mmHg, a third measurement was taken, and the average of the last two readings was used. Hypertension was defined as elevated BP, self-reported hypertension, or use of hypertensive medications. Severe hypertension was defined as SBP exceeding 160 mmHg or diastolic BP (DBP) exceeding 100 mmHg (12). Antihypertensive medication included angiotensin-converting enzyme inhibitors (ACEIs) or angiotensin receptor blockers (ARBs), β-blockers, calcium channel blockers (CCBs), diuretics, traditional compound drugs, and others. Traditional compound drugs included compound reserpine triamterene, compound reserpine, compound dihydralazine sulfate, compound trizin and rutinum, and compound kendir. Others include reserpine, α-1 receptor blocker, and renin inhibitors, etc. Blood lipid tests were performed using whole blood samples with a rapid lipid analyzer (CardioChek PA Analyzer; Polymer Technology Systems, Shanghai, China).

Participants were asked to wear light clothes, no shoes, and no headwear for measurements of height and weight. Body mass index (BMI) was calculated as the weight in kilograms divided by the square of height in meters. Obesity was defined as a BMI of at least 28 kg/m^2^ in accordance with the recommendations of the Working Group on Obesity in China (13). Standardized in-person interviews were conducted by trained personnel to collect information on sociodemographic status, medical history, and medication usage. Educational level was classified into below middle school or middle school and higher, occupation into farmer or non-farmer, and marital status into married or unmarried. Smoking status and alcohol consumption were classified as current smoker or other, and current alcohol user or other. Stroke survivors was defined by self-reported history of stroke divided into IS, haemorrhagic stroke (HS), or unclassified; years since diagnosis were also recorded. Self-reported history of stroke has proven to be sensitive, specific, and reliable (14). For other medical history, diabetes was defined as self-reported history or use of antidiabetic agents. Coronary artery disease was defined as self-reported history of angina, myocardial infarction, percutaneous coronary intervention, or coronary artery bypass craft. Self-reported use of antiplatelet drugs or statins was collected. Geographic regions included northeast China, north China, east China, central China, south China, northwest China, and southwest China, classified based on the geographical divisions of China.

### Ascertainment of outcomes

As the outcome of prevalence analyses, elevated BP was defined as a mean SBP ≥140 mmHg or DBP ≥90 mmHg. In the attributed mortality analyses, the outcomes of interest were CV death and all-cause death until 31 December 2020. We ascertained the vital status of each enrolled participant through the National Mortality Surveillance System and Vital Registration of Chinese Center for Disease Control and Prevention (CDC). All events were coded using the International Classification of Diseases (ICD)-10. CV death was defined as ICD-10 from I01-I99.

### Statistical analysis

Participant characteristics were expressed as means with standard deviations (SDs) or medians with interquartile ranges (IQRs) for continuous variables where appropriate, and proportions for categorical variables. Prevalence of elevated BP was reported overall and stratified separately by age group (35–44, 45–54, 55–64, and 65–75 years), sex (male and female), urban or rural area, and geographic region. Treatment rate was reported among all stroke survivors with elevated BP. The frequency of antihypertensive medication usage among stroke survivors was calculated. Differences in continuous and categorical variables were compared using the Kruskal–Wallis test or chi-squared test, respectively. We examined prevalence of elevated BP across age groups using the Cochran–Armitage trend test. The all-cause and CV mortality by elevated BP were compared by chi-square test. We compared the cumulative incidence of all-cause death or cardiovascular with Kaplan–Meier method stratified by elevated or normal BP.

We used Cox regression models to estimate the overall, age- and sex-specific hazard ratios (HRs) and 95% confidence intervals (CIs) of exposure to elevated BP for CV death and all-cause death relative to normal BP. For CV death, we performed Fine–Gray analyses with non-CV death considered as competing risk. For analyses in the overall population, the models were adjusted for age, sex, geographic region, stroke type, history of diabetes, history of CHD, therapy of antiplatelet drugs, and therapy of statins. For age- or sex specific analyses, age or sex was not adjusted, respectively. For age- and sex- specific analyses, both age and sex were eliminated from the adjusted variables. We calculated population attributable fractions (PAF) using the formula PAF = [P(HR−1)]/[P(HR−1) + 1], where *P* is the prevalence of elevated BP in the population of interest and HR is the adjusted HR for outcomes obtained from the Cox regression models. The proportional hazard assumption of the Cox regression was checked using the scaled Schoenfeld residuals. In total, rates of missing value ranged from 0.1% (SBP) to 8.0% [low density lipoprotein cholesterol (LDL-C)]. Missing values were imputed with the mean value of the overall population. All analyses were performed using SAS 9.4 (SAS Institute Inc., Cary, NC, United States), and the maps were created using R 3.4.1.

## Results

Of the 3,820,651 participants enrolled participants from the China PEACE MPP, 91,406 stroke survivors (49.0% male) were included in the analyses, with a mean age of 62 (8) years. Baseline characteristics of the patients are shown in Table 1. The median (IQR) stroke duration was 4 (2–7) years and IS and HS accounted for 72.5 and 12.1% of strokes, respectively (Table 1). The mean SBP and DBP were 145.3 (20.7) and 84.0 (11.7) mmHg, respectively.

**TABLE 1:** Baseline characteristics of stroke survivors with elevated and normal blood pressure (BP).

Characteristics, *n* (%)	Overall (*n* = 91,406)	Elevated BP (*n* = 55,993)	Normal BP (*n* = 35,413)	*P-value*
Demographic and socioeconomic				
Age, years, mean (SD)	62 (8)	63 (8)	61 (8)	<0.0001
Age ≥ 65	38,755 (42.4)	25,049 (44.7)	13,706 (38.7)	<0.0001
Male	44,748 (49.0)	27,578 (49.3)	17,170 (48.5)	0.02
Rural area	52,516 (57.5)	33,169 (59.2)	19,347 (54.6)	<0.0001
Education: below middle school	42,907 (46.9)	27,590 (49.3)	15,317 (43.3)	<0.0001
Occupation: farmer	43,983 (48.1)	28,115 (50.2)	15,868 (44.8)	<0.0001
Marital status: married	82,090 (89.8)	50,146 (89.6)	31,944 (90.2)	0.002
Risk factors				
BMI, kg/m^2^, mean (SD)	25.4 (3.4)	25.8 (3.4)	24.8 (3.3)	<0.0001
Obesity	19,584 (21.4)	13,808 (24.7)	5,776 (16.3)	<0.0001
Current smoker	20,023 (21.9)	11,757 (21.0)	8,266 (23.3)	<0.0001
Current alcohol user	9,981 (10.9)	6,549 (11.7)	3,432 (9.7)	<0.0001
SBP, mmHg, mean (SD)	145 (21)	158 (16)	126 (10)	<0.0001
DBP, mmHg, mean (SD)	84 (12)	89 (11)	76 (8)	<0.0001
LDL, mmol/L, median (IQR)	2.3 (1.7, 3.0)	2.4 (1.8, 3.0)	2.3 (1.7, 2.9)	<0.0001
Hypertension*	53,493 (58.5)	39,583 (70.7)	13,910 (39.3)	<0.0001
Diabetes	16,452 (18.0)	10,726 (19.2)	5,726 (16.2)	<0.0001
Years since stroke diagnosis, years, median (IQR)	4 (2, 7)	4 (2, 7)	3 (1, 6)	<0.0001
Stroke subtype				
Hemorrhagic stroke	11,100 (12.1)	7,275 (13.0)	3,825 (10.8)	<0.0001
Ischemic stroke	66,291 (72.5)	40,469 (72.3)	25,822 (72.9)	0.03
Coronary heart disease	4,275 (4.7)	2,502 (4.5)	1,773 (5.0)	0.0002

*Self-reported history of hypertension. BP, blood pressure; BMI, body mass index; SBP, systolic blood pressure; DBP, diastolic blood pressure; LDL, low density lipoprotein cholesterol; SD, standard deviation; IQR, interquartile range.

### Prevalence of elevated blood pressure

**FIGURE 1:**
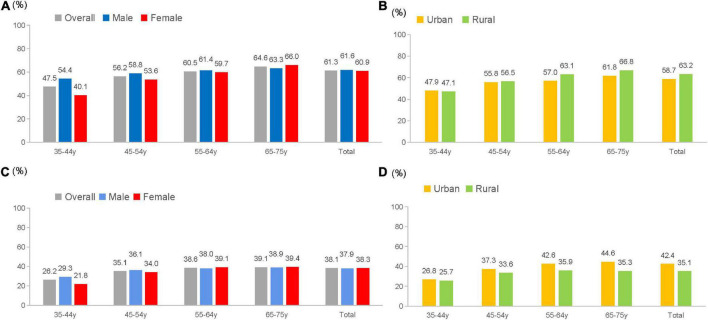
Prevalence and treatment rate of elevated blood pressure (BP) among stroke survivors. **(A)** Prevalence of elevated BP stratified by age and sex among stroke survivors. **(B)** Prevalence of elevated BP stratified by age and urbanity among stroke survivors. **(C)** Treatment rate of elevated BP stratified by age and sex among stroke survivors with elevated BP. **(D)** Treatment rate of elevated BP stratified by age and urbanity among stroke survivors with elevated BP.

**FIGURE 2:**
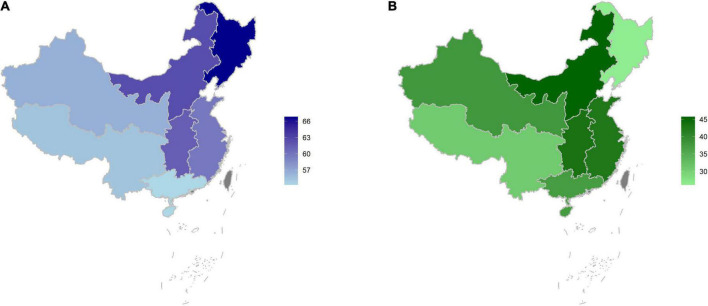
Prevalence of elevated blood pressure (BP) among stroke survivors **(A)** and treatment rate among stroke survivors with elevated BP by geographic region **(B)**.

Among all patients, 61.3% had elevated BP, and 25.7% of them had severe hypertension. Among patients with ischemic and haemorrhagic stroke, the prevalence of elevated BP was 61.1 and 65.5%, respectively. Among patients treated with antihypertensive medications, 72.5% had elevated BP. The prevalence of elevated BP stratified by age, sex, urban-rural area, and geographic region are shown in Figures 1, 2. The prevalence of elevated BP increased with age (from 47.5% at 35–44 years of age to 64.6% at 65–75 years of age) (*p*_trend_ < 0.0001) and was higher in rural area (63.2%) than in urban area (58.7%) (*p* < 0.0001). The prevalence of elevated BP did not differ considerably between sexes among all patients, while there were differences across age groups; the prevalence of elevated BP in female patients increased with age by a larger extent than in male patients. Moreover, the prevalence of elevated BP increased with age by a larger extent in patients from rural area than in those from urban area (Figure 1). Elevated BP was more prevalent in northeast (66.8%) and north China (62.4%), and less prevalent in south (54.3%) and southwest China (55.4%) (Figure 2). The prevalence of elevated BP in northwest China, central China and east China was 56.6, 61.3 and 59.9%, respectively. The baseline characteristics of patients with elevated or normal BP are shown in Table 1. Compared with patients with normal BP, those with elevated BP were older, had a lower educational level, more likely to be farmers, had higher prevalence of obesity and diabetes, and longer time since stroke. Nearly 30% of the patients with elevated BP were unaware of their hypertension status.

### Medication treatment of elevated blood pressure

Among all stroke survivors, 32.2% were treated with antihypertensive medications. The treatment rates in patients with IS and HS were 32.6 and 40.0%, respectively. For those with elevated BP, 38.1% were treated. The proportion of treated patients was low in the entire population; disparities were seen between age groups, sexes, and geographic regions. Treatment rates were similar in male and female patients across age groups and higher in patients from urban area than in those from rural area (Figure 1). Geographically, the treatment rate of elevated BP was lower in northeast China (26.1%) and southwest China (30.9%), and higher in north China (45.7%) and central China (43.5%) (Figure 2). Treatment rate in northwest China, east China and south China was 38.0, 42.8, and 37.4%, respectively. Medication treatment among stroke survivors is shown in Supplementary Table 1. Patients with elevated BP were mostly treated with one antihypertensive medication (28.9%), and less than 10% were taking two or more classes of drugs. The most used medication class was CCB (24.1%), followed by ACEI/ARB (13.8%). The most used combination antihypertensive regimen was CCB plus ACEI/ARB (4.1%) (Supplementary Table 1).

### Effect of elevated blood pressure on outcomes

After excluding participants lacking information on survival status, we included 91,333 stroke survivors in the outcome analyses. During a median of 34 (23–56) months of follow up, 3,472 (3.8%) patients died, and 2,059 (2.3%) of them died of CV causes. CV mortality and all-cause mortality were both higher in patients with elevated BP than in those with normal BP (Supplementary Figure 1, CV mortality: 2.8 vs. 1.5%, *p* < 0.0001; all-cause mortality: 4.4 vs. 2.9%, *p* < 0.0001). After adjustment for covariates, elevated BP was associated with 79 and 44% higher risk of CV death and all-cause death, respectively [CV death, HR 1.79, 95% CI (1.62–1.98), *p* < 0.0001; all-cause death: HR 1.44, 95% CI (1.34–1.55), *p* < 0.0001, Table 2]. According to the PAF, elevated BP accounted for 33 and 21% of CV deaths and all-cause deaths, respectively. Notably, elevated BP accounted for similar proportions of CV deaths across all age groups, except for a remarkably higher proportion in patients aged 35–54 years (PAF > 50%) (Table 2).

**TABLE 2:** Age- and sex-specific proportions of cardiovascular and all-cause deaths attributable to elevated blood pressure (BP).

Age	Sex	Cardiovascular death		All-cause death	
		HR (95% CI)	PAF (95% CI)	HR* (95% CI)	PAF (95% CI)
35–54	Male	2.93 (1.84–4.70)	53 (33–68)	1.57 (1.14–2.15)	25 (8–40)
	Female	3.58 (1.89–6.82)	57 (32–75)	2.20 (1.39–3.51)	38 (17–57)
	Total	3.24 (2.22–4.73)	55 (40–67)	1.79 (1.39–2.33)	30 (18–42)
55–64	Male	1.75 (1.41–2.16)	32 (20–42)	1.44 (1.23–1.68)	21 (12–29)
	Female	1.78 (1.31–2.43)	32 (16–46)	1.55 (1.24–1.94)	25 (13–36)
	Total	1.76 (1.48–2.10)	31 (23–40)	1.48 (1.30–1.69)	23 (15–29)
65–75	Male	1.69 (1.45–1.99)	30 (22–39)	1.33 (1.18–1.49)	17 (10–24)
	Female	1.58 (1.26–1.97)	28 (15–39)	1.48 (1.25–1.75)	24 (14–33)
	Total	1.56 (1.46–1.89)	27 (23–37)	1.38 (1.25–1.51)	20 (14–25)
35–75	Male	1.79 (1.58–2.02)	33 (26–39)	1.38 (1.26–1.51)	19 (14–24)
	Female	1.77 (1.49–2.11)	32 (23–40)	1.56 (1.37–1.78)	25 (18–32)
Overall		1.79 (1.62–1.98)	33 (28–38)	1.44 (1.34–1.55)	21 (17–25)

CI, confidence interval; HR, hazard ratio; PAF, population attributable fraction.

PAF = [P(HR−1)]/[P(HR−1) + 1], where *P* is the prevalence of elevated BP in the population of interest and HR is the adjusted HR for outcomes obtained from the Cox regression models.

For age- and sex- specific analyses, we adjusted for geographic regions, stroke type, history of diabetes, history of coronary heart disease, therapy of antiplatelet drugs, and therapy of statins.

For age-specific analyses, we adjusted for sex, geographic regions, stroke type, history of diabetes, history of coronary heart disease, therapy of antiplatelet drugs, and therapy of statins. For sex-specific analyses, we adjusted for age, geographic regions, stroke type, history of diabetes, history of coronary heart disease, therapy of antiplatelet drugs, and therapy of statins. For analyses in the overall population, we adjusted for age, sex, geographic regions, stroke type, history of diabetes, history of coronary heart disease, therapy of antiplatelet drugs, and therapy of statins.

## Discussion

To the best of our knowledge, this is the first study to present the prevalence and medication treatment of elevated BP among stroke survivors overall and by age group, sex, and geographic region on a national scale in China. Over 60% of stroke survivors had elevated BP, and less than 40% of them were being treated. While notable variations existed between age groups, sexes, and regions, elevated BP and its non-treatment were highly prevalent across all subgroups. Elevated BP contributed to one-third of CV deaths, accounting for over 50% of those in young and middle-aged patients, highlighting the importance for stroke management by optimizing antihypertensive treatment.

Our study extends the previous literature by performing a comprehensive descriptive analysis of BP management based on a large-scale sample of community-dwelling stroke survivors in China. While nationwide epidemiological studies on BP management among stroke survivors are available for the United States (15, 16), comparable information was limited in China, the country with the highest prevalence of stroke and stroke mortality in the world (17). Previous studies were of limited sample size with small geographic coverage, precluding further detailed analyses for age-, sex-, and region-specific trends across the country. By leveraging nearly 100,000 patients’ data from all 31 provinces in mainland China, we analyzed the prevalence of elevated BP and antihypertensive treatment among stroke survivors overall and by population subgroups, such as age, sex, and geographic regions. Moreover, our study was distinguished by further estimating the influence of suboptimal BP control on total mortality in stroke survivors. Our study provided essential information for future formulation of effective secondary prevention strategy to mitigate the growing stroke burden.

Broadly consistent with previous studies, our study demonstrated the enormously high prevalence of elevated BP in China, contrary to findings from the 2005 to 2016 National Health and Nutrition Examination Survey in the United States, where 37.1% of stroke survivors had elevated BP (15). Moreover, our study identified different distributions of elevated BP across age groups, sexes, and geographic regions. Our study found discrepancies in the prevalence of elevated BP across age groups and urban-rural areas, consistent with similar patterns in the prevalence of hypertension in the general population. The prevalence of elevated BP increased by a larger extent in female patients as well as those from rural area, which was also discovered in the general population (18), possibly reflecting the underlying role of menopause and socioeconomic factors in elevated BP. Furthermore, our study also revealed a north-to-south gradient for elevated BP prevalence with the greatest burden in north and central regions, aligning with the regional pattern of stroke burden in previous reports (17, 19). This convergence is likely the synergistic effect of the detrimental role of BP elevation in the development and recurrence of stroke, underscoring the unmet need in optimizing BP management to reduce stroke burden.

Our findings exhibited treatment gaps and highlighted substantial opportunities to initiate, intensify or improve the antihypertensive treatment among stroke survivors. Less than 40% of patients with elevated BP were treated, and most were treated with only one single antihypertensive medication, similar to the treatment rate (37.7%) and single-pill prescription pattern reported in a survey of patients from secondary and tertiary hospitals in 2008 (8). These rates were far behind the findings in the United States, where over 80% of stroke survivors with elevated BP were treated, and 56% of them with combination therapy (15). The disparity was intensified regarding the situation in northeast China, which had the highest prevalence of elevated BP and lowest treatment rate, which partially explains the apparently heavy stroke burden in this region (18, 20, 21). Of note, the choice of hypertensive medications for elevated BP in stroke survivors differed in China and the United States. CCB was the most used medication among stroke survivors in China, compared with ACEI/ARB in the United States (17). While the optimal choice of antihypertensive medications remains to be elucidated, pooled analyses from randomized controlled trials supports the use of diuretics and ACEI for secondary prevention of stroke (20, 22). Although CCBs have been preferentially recommended for the primary prevention of stroke (21, 23), their comparable effectiveness relative to other medication classes in the secondary prevention of stroke warrants further examination. Improved use of diuretics is another future intervention direction.

The importance of our study was strengthened by estimating the influence of elevated BP on the long-term prognosis of stroke survivors with PAF. One-third of CV deaths were attributed to the poor management of BP. Furthermore, as indicated by age-specific PAF, our study revealed a higher-than-expected contribution of elevated BP to CV death among young and middle-aged patients. While the underlying reason for this disparity remains unclear, our study corroborated the benefits of lifelong BP management in stroke survivors of all ages. This is especially important for younger stroke patients who remain at substantial risk of CV events for decades (24, 25). Higher death burden due to elevated BP among younger stroke survivors underscores the importance of early and intensive intervention strategy.

Along with emerging evidence of suboptimal uptake of secondary prevention of CV diseases in China (26–28), our findings have strong clinical and public health implications. Given the increasing prevalent strokes and stroke-related deaths in China and other developing countries, our study greatly informs the formulation of intervention strategy to improve antihypertensive treatment among stroke survivors. Widely accessible and cost-effective strategies to cover post-discharge care are required to improve BP management and other secondary prevention measures in stroke survivors on the system, provider, and patient levels (29). The introduction of strong incentives to improve both patient and physician behavior through a team-based approach by involving physicians, nurses, and other ancillary staff has been suggested, which could result in more careful and frequent follow-up and definitive control of stroke risk factors. It would be ideal and feasible to improve adherence in primary health settings through the support of appropriate and readily available referrals and services (30). Since the potential for improving secondary prevention among primary care physicians persists (31), implementation strategies for physician training are required to enhance guideline adherence, especially improving clinical inertia regarding the BP management on provider’s part. Moreover, emerging novel techniques, including telemedicine and mobile health can be leveraged to enhance patients’ awareness and self-management capability among stroke survivors (32, 33).

The results of our study should be interpreted considering the following limitations. First, stroke in this study was determined by self-report without validation, although self-reported stroke has proven to be sensitive, specific, and reliable. Our study also did not collect the information regarding the status of repeated stroke, as well as combination of ischemic and hemorrhagic stroke at the baseline, which may have different influence on the outcomes. Second, the results of the study were obtained from a convenience sample and should be generalized with caution. However, to the best knowledge, our study was the largest to assess BP management among stroke survivors recruited from geographically diverse regions covering all 31 provinces in mainland China. Third, our study sample was screened from community-dwelling participants; patients with functional disability were less likely to be recruited and were likely underrepresented. Fourth, our study did not assess the effect of elevated BP on stroke recurrence or other CV events, which are also important outcomes of interest.

## Conclusion

In this nationwide sample of stroke survivors, over 60% had elevated BP, and less than 40% of them were treated. BP management was suboptimal among all stroke survivors, irrespective of age, sex, and region. Elevated BP was associated with 79% higher risk of CV death and accounted for a third of CV mortality and more than 50% in young and middle-aged patients. National strategies are required to improve the management of elevated BP in the secondary prevention of stroke to mitigate the increasing stroke burden in China.

## Data availability statement

The data are not publicly available. The China Patient-Centered Evaluative Assessment of Cardiac Events Million Persons Project only provides conditional data access for qualified researchers with legitimate requests. A formal application and research proposal is required. Please contact cvd-project@nccd.org.cn to seek approval for data access.

## Ethics statement

The studies involving human participants were reviewed and approved by the Central Ethics Committee at the China National Center for Cardiovascular Diseases. All enrolled participants provided written informed consent.

## Author contributions

JLu and JCa conceived the study and took responsibility for all aspects of it. JLu, JCa, and BW designed the study. BW wrote the first draft of the manuscript with further contributions from XB, YY, JCu, LS, JLi, JLu, and JCa. BW and XB did the statistical analysis. BW, JLu, and XB had access to the raw data. All authors interpreted the data and approved the final version of the manuscript.

## References

[1] GBD 2016 Causes of Death Collaborators. Global, regional, and national age-sex specific mortality for 264 causes of death, 1980-2016: a systematic analysis for the global burden of disease study 2016. *Lancet.* (2017) 390:1151–210. 28919116 10.1016/S0140-6736(17)32152-9 PMC5605883

[2] GBD 2016 DALYs and HALE Collaborators. Global, regional, and national disability-adjusted life-years (dalys) for 333 diseases and injuries and healthy life expectancy (hale) for 195 countries and territories, 1990-2016: a systematic analysis for the global burden of disease study 2016. *Lancet.* (2017) 390:1260–344. 28919118 10.1016/S0140-6736(17)32130-X PMC5605707

[3] The Writing Committee of the Report on Cardiovascular Health and Diseases in China. Report on cardiovascular health and diseases burden in china: an updated summary of 2020. *Chin Circ J.* (2021) 36:521–45.

[4] GBD 2016 Stroke Collaborators. Global, regional, and national burden of stroke, 1990-2016: a systematic analysis for the global burden of disease study 2016. *Lancet Neurol.* (2019) 18:439–58. 30871944 10.1016/S1474-4422(19)30034-1 PMC6494974

[5] PROGRESS Collaborative Group. Randomised trial of a perindopril-based blood-pressure-lowering regimen among 6105 individuals with previous stroke or transient ischaemic attack. *Lancet.* (2001) 358:1033–41. 10.1016/S0140-6736(01)06178-5 11589932

[6] Katsanos AH Filippatou A Manios E Deftereos S Parissis J Frogoudaki A Blood pressure reduction and secondary stroke prevention: a systematic review and metaregression analysis of randomized clinical trials. *Hypertension.* (2017) 69:171–9. 10.1161/HYPERTENSIONAHA.116.08485 27802419

[7] Liu L Wang D Wong KS Wang Y. Stroke and stroke care in china: huge burden, significant workload, and a national priority. *Stroke.* (2011) 42:3651–4. 10.1161/STROKEAHA.111.635755 22052510

[8] Wang Y Wu D Zhou Y Zhao X Wang C Liu L Survey of blood pressure control status in patients with ischemic stroke or transient ischemic attack in china. *Neurol Res.* (2008) 30:348–55. 10.1179/174313208X300323 18544250

[9] Xiujun M Shuai H Xueping F Zhiwei K Sulan G Ruizhen X Status of management of essential hypertension for patients with ischemic stroke in basal medical institutions of liaoning province. *J Apoplexy Nervous Dis.* (2010) 27:544–8.

[10] Huijuan Z Chenghuan W Dong Y Dianhai F Juan Z Yun L. Current situation of secondary prevention and risk factors related to recurrent stroke in patients with ischemia stroke in communities. *Chin J Evid Based Cardiovasc Med.* (2014) 6:80–3.

[11] Lu J Xuan S Downing NS Wu C Li L Krumholz HM Protocol for the china peace (patient-centered evaluative assessment of cardiac events) million persons project pilot. *BMJ Open.* (2016) 6:e010200. 10.1136/bmjopen-2015-010200 26729395 PMC4716208

[12] China PEACE MPP Collaborative Group. Severe hypertension in china: results from the china peace million persons project. *J Hypertens.* (2021) 39:461–70. 10.1097/HJH.0000000000002675 33038086 PMC7928212

[13] Zhou BF Cooperative Meta-Analysis Group of the Working Group on Obesity in China. Predictive values of body mass index and waist circumference for risk factors of certain related diseases in chinese adults–study on optimal cut-off points of body mass index and waist circumference in chinese adults. *Biomed Environ Sci.* (2002) 15:83–96. 12046553

[14] O’Mahony PG Dobson R Rodgers H James OF Thomson RG. Validation of a population screening questionnaire to assess prevalence of stroke. *Stroke.* (1995) 26:1334–7. 10.1161/01.STR.26.8.1334 7631332

[15] Santos D Dhamoon MS. Trends in antihypertensive medication use among individuals with a history of stroke and hypertension, 2005 to 2016. *JAMA Neurol.* (2020) 77:1382–9. 10.1001/jamaneurol.2020.2499 32716495 PMC7385676

[16] Kesarwani M Perez A Lopez VA Wong ND Franklin SS. Cardiovascular comorbidities and blood pressure control in stroke survivors. *J Hypertens.* (2009) 27:1056–63. 10.1097/HJH.0b013e32832935ce 19405168

[17] Wang W Jiang B Sun H Ru X Sun D Wang L Prevalence, incidence, and mortality of stroke in china. *Circulation.* (2017) 135:759–71. 10.1161/CIRCULATIONAHA.116.025250 28052979

[18] The China PEACE Collaborative Group. Association of age and blood pressure among 3.3 million adults: insights from china peace million persons project. *J Hypertens.* (2021) 39:1143–54. 10.1097/HJH.0000000000002793 33967218

[19] He J Klag MJ Wu Z Whelton PK. Stroke in the people’s republic of china. I. Geographic variations in incidence and risk factors. *Stroke.* (1995) 26:2222–7. 10.1161/01.STR.26.12.2222 7491640

[20] Rashid P Leonardi-Bee J Bath P. Blood pressure reduction and secondary prevention of stroke and other vascular events: a systematic review. *Stroke.* (2003) 34:2741–8. 10.1161/01.STR.0000092488.40085.15 14576382

[21] Rothwell PM Howard SC Dolan E O’Brien E Dobson JE Dahlof B Effects of beta blockers and calcium-channel blockers on within-individual variability in blood pressure and risk of stroke. *Lancet Neurol.* (2010) 9:469–80. 10.1016/S1474-4422(10)70066-1 20227347

[22] Zonneveld TP Richard E Vergouwen MD Nederkoorn PJ de Haan R Roos YB Blood pressure-lowering treatment for preventing recurrent stroke, major vascular events, and dementia in patients with a history of stroke or transient ischaemic attack. *Cochrane Database Syst Rev.* (2018) 7:CD007858. 10.1002/14651858.CD007858.pub2 30024023 PMC6513249

[23] Verdecchia P Reboldi G Angeli F Gattobigio R Bentivoglio M Thijs L Angiotensin-converting enzyme inhibitors and calcium channel blockers for coronary heart disease and stroke prevention. *Hypertension.* (2005) 46:386–92. 10.1161/01.HYP.0000174591.42889.a2 16009786

[24] Rutten-Jacobs LCA Arntz RM Maaijwee NAM Schoonderwaldt HC Dorresteijn LD van Dijk EJ Cardiovascular disease is the main cause of long-term excess mortality after ischemic stroke in young adults. *Hypertension.* (2015) 65:670–5. 10.1161/HYPERTENSIONAHA.114.04895 25624336

[25] Rutten-Jacobs LCA Arntz RM Maaijwee NAM Schoonderwaldt HC Dorresteijn LD van Dijk EJ Long-term mortality after stroke among adults aged 18 to 50 years. *JAMA-J Am Med Assoc.* (2013) 309:1136–44. 10.1001/jama.2013.842 23512060

[26] Yusuf S Islam S Chow CK Rangarajan S Dagenais G Diaz R Use of secondary prevention drugs for cardiovascular disease in the community in high-income, middle-income, and low-income countries (the pure study): a prospective epidemiological survey. *Lancet.* (2011) 378:1231–43. 10.1016/S0140-6736(11)61215-4 21872920

[27] Chen YP Li LM Zhang QL Clarke R Chen JS Guo Y Use of drug treatment for secondary prevention of cardiovascular disease in urban and rural communities of china: china kadoorie biobank study of 0.5 million people. *Int J Cardiol.* (2014) 172:88–95. 10.1016/j.ijcard.2013.12.065 24461961 PMC3991854

[28] Lu J Zhang L Lu Y Su M Li X Liu J Secondary prevention of cardiovascular disease in china. *Heart.* (2020) 106:1349–56. 10.1136/heartjnl-2019-315884 31980439

[29] Holloway RG Benesch C Rush SR. Stroke prevention: narrowing the evidence-practice gap. *Neurology.* (2000) 54:1899–906. 10.1212/WNL.54.10.1899 10822426

[30] Kohok DD Sico JJ Baye F Myers L Coffing J Kamalesh M Post-stroke hypertension control and receipt of health care services among veterans. *J Clin Hypertens.* (2018) 20:382–7. 10.1111/jch.13194 29397583 PMC8031130

[31] Chen C Qiao X Kang H Ding L Bai L Wang J. Community physicians’ knowledge of secondary prevention after ischemic stroke: a questionnaire survey in shanxi province, china. *BMC Med Educ.* (2015) 15:197. 10.1186/s12909-015-0481-4 26530114 PMC4632687

[32] Lv M Wu T Jiang S Chen W Zhang J. Effects of telemedicine and mhealth on systolic blood pressure management in stroke patients: systematic review and meta-analysis of randomized controlled trials. *JMIR Mhealth Uhealth.* (2021) 9:e24116. 10.2196/24116 34114961 PMC8235282

[33] Owolabi MO Gebregziabher M Akinyemi RO Akinyemi JO Akpa O Olaniyan O Randomized trial of an intervention to improve blood pressure control in stroke survivors. *Circ Cardiovasc Qual Outcomes.* (2019) 12:e005904. 10.1161/CIRCOUTCOMES.119.005904 31805787 PMC7139215

