# Supplementary material for: Prevalence, treatment, and attributed mortality of elevated blood pressure among a nationwide population-based cohort of stroke survivors in China
Source: Front Cardiovasc Med. 2022 Sep 30;9:890080. doi: 10.3389/fcvm.2022.890080 (PMC9561361; doi:10.3389/fcvm.2022.890080)
Supplement: Supplementary file 1 [file Table_1.docx]

**Supplemental Material**

**Table S1** Medication treatment among stroke survivors

| Medication treatment, n (%) | **Overall**  **(n=91,406)** | **Elevated BP**  **(n=55,993)** | **Normal BP**  **(n=35,413)** |
| --- | --- | --- | --- |
| Antihypertensive medication |  |  |  |
| ACEI | 3,847 (4.2) | 2,977 (5.3) | 870 (2.5) |
| ARB | 7,046 (7.7) | 4,842 (8.6) | 2,240 (6.2) |
| ACEI/ARB | 10,760 (11.8) | 7,718 (13.8) | 3,042 (8.6) |
| β-blocker | 2,816 (3.1) | 1,904 (3.4) | 912 (2.6) |
| CCB | 18,585 (20.3) | 13,509 (24.1) | 5,076 (14.3) |
| Diuretics | 3,031 (3.3) | 2,131 (3.8) | 900 (2.6) |
| Traditional compound drugs | 1,850 (2.0) | 1,406 (2.5) | 444 (1.3) |
| Other | 711 (0.8) | 548 (1.0) | 163 (0.5) |
| No. of antihypertensive medications |  |  |  |
| None | 61,964 (67.8) | 34,665 (61.9) | 27,299 (77.1) |
| 1 | 22,237 (24.3) | 16,197 (28.9) | 6,040 (17.1) |
| ≥2 | 7,205 (7.9) | 5,131 (9.2) | 2,074 (5.9) |
| Dual antihypertensive medication |  |  |  |
| ACEI/ARB+CCB | 3,177 (3.5) | 2,293 (4.1) | 884 (2.5) |
| ACEI/ARB+β-blocker | 381 (0.4) | 259 (0.5) | 122 (0.3) |
| ACEI/ARB+Diuretics | 844 (0.9) | 555 (1.0) | 289 (0.8) |
| β-blocker+Diuretics | 59 (0.1) | 43 (0.1) | 16 (<0.1) |
| Triple antihypertensive medication |  |  |  |
| ACEI/ARB+CCB+Diuretics | 415 (0.5) | 292 (0.5) | 123 (0.3) |
| ACEI/ARB+β-blocker+CCB | 342 (0.4) | 231 (0.4) | 111 (0.3) |
| ACEI/ARB+β-blocker+Diuretics | 90 (0.1) | 60 (0.1) | 30 (0.1) |
| β-blocker+CCB+Diuretics | 22 (<0.1) | 15 (<0.1) | 7 (<0.1) |
| Antiplatelet | 13,498 (14.8) | 8,237 (14.7) | 5,261 (14.9) |
| Statin | 8,120 (8.9) | 4,717 (8.4) | 3,403 (9.6) |

ACEI: angiotensin converting enzyme inhibitor; ARB: angiotensin receptor blocker; CCB: calcium channel blocker.

**Figure S1** Kaplan Meier curve of all-cause death or cardiovascular (CV) death stratified by elevated or normal blood pressure.
