# Supplementary figures and images for: Prevalence, treatment, and attributed mortality of elevated blood pressure among a nationwide population-based cohort of stroke survivors in China
Source: Front Cardiovasc Med. 2022 Sep 30;9:890080. doi: 10.3389/fcvm.2022.890080 (PMC9561361; doi:10.3389/fcvm.2022.890080)

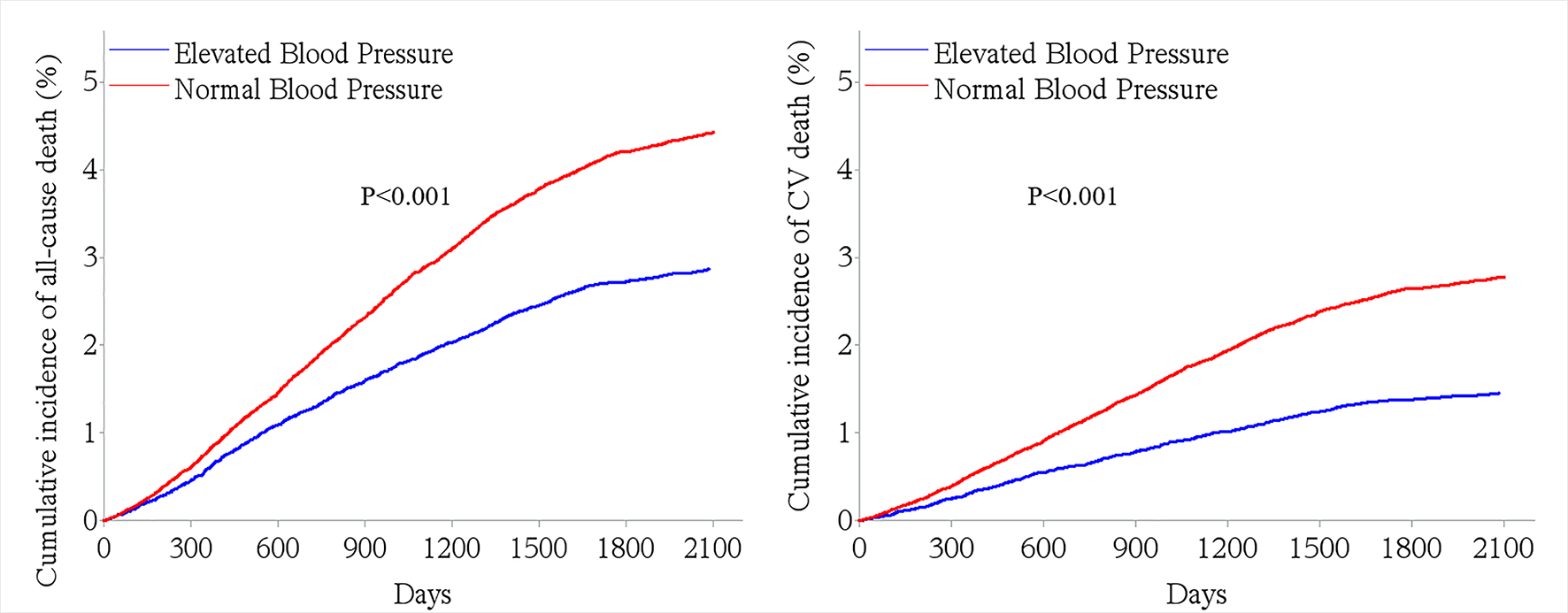

Supplement: Supplementary file 2 [file Image_1.tif]
